# Supplementary material for: Prognostic implications of metabolism-associated gene signatures in colorectal cancer
Source: PeerJ. 2020 Sep 2;8:e9847. doi: 10.7717/peerj.9847 (PMC7474523; doi:10.7717/peerj.9847)
Supplement: Table S1 [file peerj-08-9847-s003.docx]

**Supplementary Table 1.** Characteristics of TCGA colorectal cancer cohort and GEO dataset (tumor).

|  |  | **TCGA** | **GEO** |
| --- | --- | --- | --- |
| **Age(years)** |  | 66.20±12.85 | 65.55±12.69 |
| **Gender** | Female | 281 | 301 |
|  | Male | 315 | 419 |
| **Stage** | Stage Ⅰ | 107 | 31 |
|  | Stage Ⅱ | 223 | 311 |
|  | Stage Ⅲ | 177 | 309 |
|  | Stage Ⅳ | 89 | 69 |
| **T (Tumor)** | T1 | 18 | 11 |
|  | T2 | 105 | 48 |
|  | T3 | 407 | 535 |
|  | T4 | 66 | 126 |
| **N (Lymph Node)** | N0 | 354 | 356 |
|  | N1 | 150 | 261 |
|  | N2 | 116 | 97 |
|  | N3 | 0 | 6 |
| **M (Metastasis)** | M0 | 455 | 650 |
|  | M1 | 87 | 70 |
|  | Mx | 54 | 68 |
