# Supplementary material for: Prognostic implications of metabolism-associated gene signatures in colorectal cancer
Source: PeerJ. 2020 Sep 2;8:e9847. doi: 10.7717/peerj.9847 (PMC7474523; doi:10.7717/peerj.9847)
Supplement: Table S2 [file peerj-08-9847-s004.docx]

**Supplementary Table 2** The correlation between the expression of genes and the clinicopathological features of TCGA-CRC

| **Id** | **Age (P-value)** | **Gender (P-value)** | **Stage (P-value)** | **T (P-value)** | **M (P-value)** | **N (P-value)** |
| --- | --- | --- | --- | --- | --- | --- |
| AKR1C4 | -4.364(1.501e-05) | -3.453(5.898e-04) | -3.111(0.002) | -4.54(1.89e-05) | -3.382(7.628e-04) | 1.862(0.066) |
| SPHK1 | -4.101(4.665e-05) | -2.892(0.004) | -5.209(2.554e-07) | -4.516(2.257e-05) | -5.514(5.147e-08) | 1.531(0.130) |
| GPX3 | -1.311(0.190) | 0.942(0.347) | -2.719(0.007) | -3.808(2.87e-04) | -2.485(0.013) | -1.756(0.084) |
| NAT2 | -3.717(2.19e-04) | -5.22(2.449e-07) | -1.115(0.265) | -0.248(0.805) | -1.434(0.152) | 2.818(0.006) |
| XDH | -5.45(7.366e-08) | -3.771(1.77e-04) | -2.53(0.012) | -4.192(6.815e-05) | -2.965(0.003) | 2.778(0.007) |
| ADCY5 | 3.431(6.439e-04) | 1.65(0.100) | 5.209(2.623e-07) | 3.519(6.602e-04) | 5.399(9.802e-08) | -2.025(0.046) |
| riskScore | 3.951(8.649e-05) | 4.258(2.403e-05) | 1.701(0.089) | 0.495(0.622) | 2.126(0.034) | -3.285(0.002) |
