# Supplementary material for: Prognostic implications of metabolism-associated gene signatures in colorectal cancer
Source: PeerJ. 2020 Sep 2;8:e9847. doi: 10.7717/peerj.9847 (PMC7474523; doi:10.7717/peerj.9847)
Supplement: Table S3 [file peerj-08-9847-s005.docx]

**Supplementary Table 3** The correlation between the expression of genes and the clinicopathological features of GEO-CRC

| **Id** | **Age (P-value)** | **Gender (P-value)** | **Stage (P-value)** | **T (P-value)** | **M (P-value)** | **N (P-value)** |
| --- | --- | --- | --- | --- | --- | --- |
| AKR1C4 | 1.804(0.072) | -1.054(0.292) | -1.091(0.276) | 0.483(0.630) | -1.003(0.319) | -1.265(0.207) |
| SPHK1 | -0.811(0.418) | 1.547(0.123) | -0.556(0.578) | -3.489(5.669e-04) | 0.376(0.708) | -1.022(0.307) |
| GPX3 | -1.57(0.117) | 1.402(0.162) | -1.496(0.136) | -2.313(0.021) | -1.003(0.319) | -1.756(0.080) |
| NAT2 | 0.269(0.788) | 0.45(0.653) | 3.28(0.001) | 1.742(0.084) | 2.341(0.021) | 3.028(0.003) |
| XDH | 1.962(0.050) | 0.747(0.456) | 1.69(0.092) | 0.645(0.520) | 1.523(0.130) | 1.705(0.089) |
| ADCY5 | -1.179(0.239) | 2.599(0.010) | -2.912(0.004) | -0.37(0.712) | -0.978(0.330) | -2.78(0.006) |
| riskScore | -1.424(0.155) | 0.715(0.475) | -4.046(6.171e-05) | -2.104(0.037) | -2.541(0.013) | -4.047(6.197e-05) |
