# Supplementary material for: Prognostic implications of metabolism-associated gene signatures in colorectal cancer
Source: PeerJ. 2020 Sep 2;8:e9847. doi: 10.7717/peerj.9847 (PMC7474523; doi:10.7717/peerj.9847)
Supplement: Figure S1 — Heatmaps (A) and volcano diagram (B) show the differentially expressed metabolic genes. higher expression manifested in red; lower expressions were green FDR < 0.05, logFC > 1.5. [file peerj-08-9847-s001.pdf]

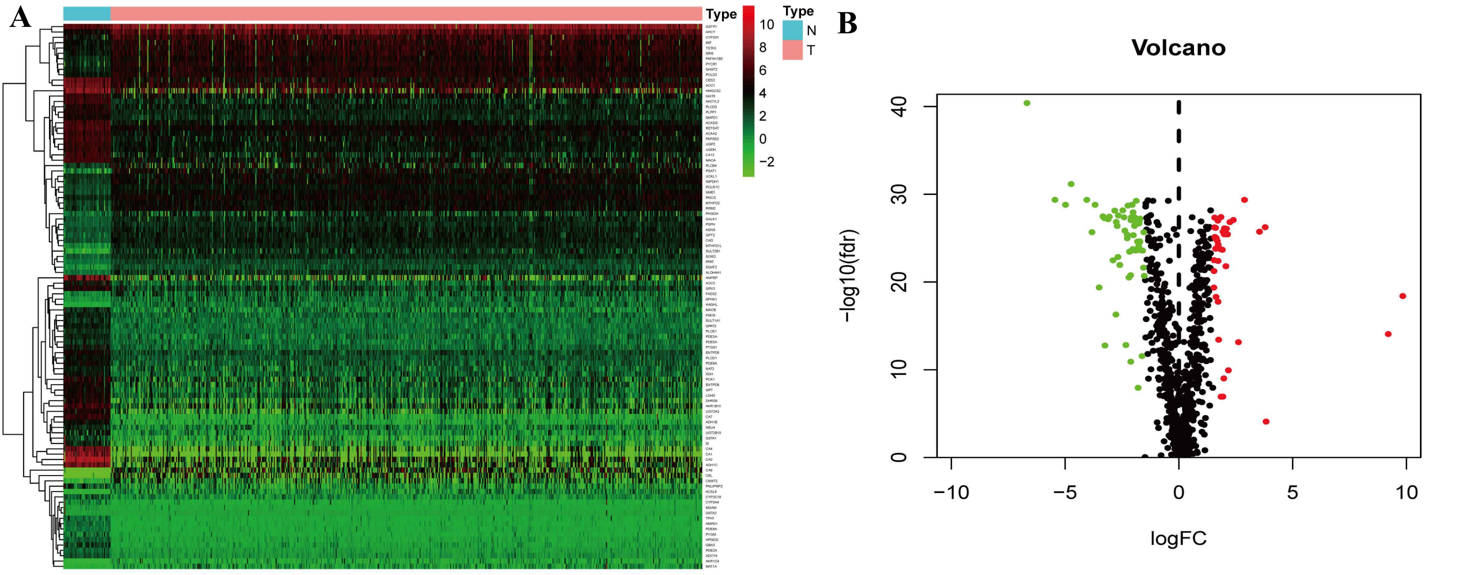
A
Type
10
8
6
4
2
0
−2
Type
N
T
B
Volcano
−log10(fdr)
40
30
20
10
0
−10
−5
0
5
10
logFC
